# Parental Stress Experience and Age of Mothers and Fathers After Preterm Birth and Admission of Their Neonate to Neonatal Intensive Care Unit; A Prospective Observational Pilot Study

**DOI:** 10.3389/fped.2019.00439

**Published:** 2019-10-24

**Authors:** Elisabeth Pichler-Stachl, Pia Urlesberger, Christian Mattersberger, Nariae Baik-Schneditz, Berndt Schwaberger, Berndt Urlesberger, Gerhard Pichler

**Affiliations:** Division of Neonatology, Department of Pediatrics, Medical University of Graz, Graz, Austria

**Keywords:** parental stress, preterm birth, mother, father, age

## Abstract

**Background:** Preterm birth is associated with increased stress of parents that might influence the parental-child interaction, thus potentially having influence on the neurobehavioral development of the preterm infants. However, little is known concerning the age dependency of parental stress after preterm birth.

**Objective:** The aim of the present study was to examine the age dependency of stress in mothers and fathers after preterm birth and neonatal intensive care unit (NICU) admission of their infant.

**Methods:** In a prospective observational pilot study 47 mothers and 47 fathers completed the parental stress scale:NICU (PSS:NICU) questionnaire within 72 h after delivery. This questionnaire measures parental stress after preterm birth with three subscales: “Looks and Behave” of the child, “Parental Role Alteration,” and “Sights and Sounds.” Stress levels of mothers and fathers were compared and correlated to the age of mothers and fathers, respectively.

**Results:** Parental stress experience after preterm birth tended to be higher in mothers compared to fathers. Mothers showed a significant positive correlation of the “Sights and Sounds” scale and age, whereas fathers did not show any significant age dependency of stress.

**Conclusion:** In mothers stress level increases with increasing maternal age after preterm birth and admission of their infant to NICU, whereas fathers did not show any significant age dependency of stress.

## Introduction

Preterm birth is frequently associated with increased parental stress, worry, and anxiety, during and after admission to a Neonatal Intensive Care Unit (NICU) (1–3). Mothers of former preterm infants report greater concerns about their children's health outcomes, less consensus with partners, and less years of education attained than mothers of term infants (1).

Miles et al. (4) developed a questionnaire to quantify parental stress in the particular situation of preterm birth, the Parental Stress Scale: Neonatal Intensive Care Unit (PSS:NICU), which contains three subscales: “Sights and Sound,” “Parental Role Alteration,” and “Looks and Behave” of the child (4).

Reports on the stress experience of fathers after preterm birth vary (5–7). Frank et al. reported that all aspects of giving birth to a preterm child are more stressful for mothers compared to fathers resulting in higher stress levels at all subscales of the PSS:NICU (5). In contrary Matricardi et al. did show that mothers of preterm neonates experience more stress than fathers only in regard to “Parental Role Alteration” subscale (6). One reason for the different findings might be that mothers and fathers have different stress experiences at different ages.

Therefore, the aim of the present study was to investigate, whether there is an association of the parents' age and the parental stress after preterm birth and admission of their neonate to a NICU and whether there is a difference between mothers and fathers. We hypothesized that there is an age dependency in parental stress after preterm birth in mothers and fathers, which may explain these differences.

## Methods

### Participants

A prospective observational pilot study was conducted at the Division of Neonatology at the Medical University of Graz. Parents—mothers and fathers—of preterm neonates born before 37 weeks of gestation and admitted to the NICU were eligible for this study. The study was approved by the Ethical Committee of the Medical University of Graz (EK number: 25-529 ex 12/13) and parents were included after giving written informed consent to participate in the study. Participants were given a socio-demographic questionnaire and the PSS:NICU measuring parental stress within 72 h after the birth of their child and were asked to complete it within 24 h. Parents had to be literate in German and over 18 years old. Fathers had joined the birth in the delivery room and parents were allowed to visit their neonate at any time, after the neonates were admitted to NICU.

### Sample Size

For the present prospective observational study a sample size of 50 mothers and 50 fathers was considered to be convenient (7).

### Socio-demographic Questionnaire

The following socio-demographic data were collected: parental age, educational status, marital status, and mode of delivery.

#### PSS:NICU

The PSS:NICU measures parental stress after preterm birth and admission of their neonate to NICU. It is a self-report questionnaire, which can be rated on a five point Likert-Scale: 1 = not at all stressful: the experience did not cause you to feel upset, tense, or anxious, 2 = a little stressful, 3 = moderately stressful, 4 = very stressful, and 5 = extremely stressful. Items describing situations that have not been experienced by the parents can also be answered with “not applicable.” The parents should indicate how much experiences were stressful in the subscale “Sights and Sound” consisting of 5 items (e.g., “The presence of monitors and equipment”), the subscale “Parental Role Alteration” consisting of 14 items (e.g., “Being separated from my baby”) and the subscale, “Looks and Behave” of the child (e.g., “Tubes and equipment on or near my baby”) (4).

### Statistical Analysis

Data of mothers and fathers are presented as mean and standard deviation for normally distributed continuous variables and median (interquartile range, IQR) when the distribution was skewed.

Parental age and stress levels of mothers and fathers were compared using *t*-Test or Mann–Whitney *U*-test, as appropriate. The association between parental age and stress levels in mothers and fathers were analyzed using Spearman's rank correlation coefficient or Pearson's correlation when appropriate. A *p*-value < 0.05 was considered statistical significant. The statistical analyses were performed using IBM SPSS Statistics 22.0.0 (IBM Corporation; Armonk, USA).

## Results

One hundred parents (50 mothers and 50 fathers) participated in this prospective observational study. The PSS:NICU questionnaires of 94 parents (47 mothers and 47 fathers) were included into analyses. Six parents were excluded, since the questionnaires were not completed correctly. The mothers stated their highest level of education as following: 16% compulsory education, 32% apprenticeship, 34% higher school certificate, and 18% university degree. The fathers stated their highest level of education as following: 12% compulsory education, 36% apprenticeship, 26% higher school degree, and 26% university degree. Forty four percent of the couples were married. Of the participating women 33 delivered by cesarean section and 14 women delivered vaginally. Ten women gave birth to twins. The neonates were born prematurely due to preterm prelabour rupture of membranes (*n* = 18), preterm labor (*n* = 8), vaginal bleeding (*n* = 7), eclampsia (*n* = 6), intrauterine growth retardation (*n* = 3), others (*n* = 5).

The mean age of mothers and fathers was 31 ± 5 and 33 ± 6 years, respectively (*p* = 0.07). The mean gestational age of the preterm infants was 32 ± 2 weeks of gestation and the mean birth weight was 1,714 ± 456 grams. The preterm neonates of 20 pairs of parents received respiratory support, at the time point when the questionnaires were completed.

Analyses of the PSS:NICU showed that “Parental Role Alteration” is the most stressful part for parents in the situation of a preterm birth compared to “Sights and Sounds” (*p* < 0.001) and “Looks and Behave” (*p* < 0.001). In addition, the results showed that mothers had non-significant trends to higher stress levels than fathers in the subscale “Parental Role Alteration” (Table 1).

**Table 1:** Parental stress in mothers and fathers after preterm birth assessed with the PSS:NICU questionnaire after admission of the neonate to a neonatal intensive care unit.

	**Mothers**		**Fathers**		
	**Mean**	***SD***	**Mean**	***SD***	***p*-value**
Sights and sounds	1.86	0.79	1.74	0.77	0.352
Looks and behave	1.57	1.02	1.65	0.90	0.508
Parental role	3.29	1.22	2.84	1.24	0.095
Total score	2.09	0.90	1.99	0.80	0.555

Correlation analyses demonstrated that stress correlated positively with age in mothers, but only within the subscale “Sights and Sounds” correlation was significant. In contrast, in fathers all stress subscales showed a non-significant negative correlation with age (Table 2).

**Table 2:** Correlation of parental stress and age in mothers and fathers after preterm birth assessed with the PSS:NICU questionnaire after admission of the neonate to a neonatal intensive care unit.

	**Mothers**		**Fathers**	
	**Coefficient**	***p*-value**	**Coefficient**	***p*-value**
Sights and sounds	0.29	0.048^*^	−0.03	0.841
Looks and behave	0.211	0.136	−0.212	0.153
Parental role	0.035	0.814	−0.079	0.599
Total score	0.187	0.209	−0.122	0.415

* *Significant correlation*.

## Discussion

The present study is the first analyzing gender related age dependency of parental stress after preterm birth and admission of the preterm neonate to a NICU. There was a positive correlation of stress levels and age in mothers, whereas we did not observe any significant correlation of stress levels and age in fathers. In contrary, there was a clear trend to negative correlation of stress levels and age in fathers.

In recent studies higher stress levels in mothers than in fathers have been demonstrated (5, 6, 8, 9), whereby the stress levels differed only to a small extent. In the present study we only observed a trend to higher stress in mothers compared to fathers. These findings of low overall differences in stress after preterm birth between mothers and fathers might be due to different age dependency observed in the present study.

For parents after preterm birth of their infant “Parental Role Alteration” proofed to be the highest stressor in the present study that is in accordance with the available literature (8, 9). The “Total Score” reflects the parent's general experience of being confronted with a preterm infant. Parental stress levels after preterm birth have been considered to be moderately stressful (9), whereby in former studies possible age dependencies were not considered. The present study suggests that especially in older mothers the stress level might be substantial.

Older mothers are facing from beginning of their pregnancy an overall increased risk. Therefore, we can hypothesize that this might be the reason for the present observation of increasing stress levels with increasing age after preterm birth.

Main limitation of the present study is the sample size of mothers and fathers at different ages. However, we demonstrated a significant association of age and stress in mothers. Furthermore, the different subscales of the PSS:NICU have to be interpreted with caution due to the psychometric properties and the dimensionality of the PSS: NICU (10). However, this questionnaire is widely used and accepted since its first description more than 20 years ago (4, 9).

## Conclusion

In mothers stress level increases with increasing age after preterm birth and admission of their infant to NICU, whereas fathers did not show any significant age dependency of stress.

Previous research showed that parental stress has been associated with differences in neurobehavioral development of preterm infants (11). Furthermore, integration of parents into the care of their preterm neonate at the NICU is increasing (12) that might influence parental stress. Therefore, the present findings of parental age dependent stress levels have to be further evaluated and taken into account when parents are supported after preterm birth of their infant.

## Data Availability Statement

The datasets generated for this study are available on request to the corresponding author.

## Ethics Statement

This study was carried out in accordance with the recommendations of Regional Committee on Biomedical Research Ethics with written informed consent from all subjects. All subjects gave written informed consent in accordance with the Declaration of Helsinki. The protocol was approved by the Ethikkommission, Medizinische Universität Graz, Austria.

## Author Contributions

E-PS, GP, and BU: conception and design. E-PS PU, NB-S, BS, and GP: collection and assembly of data. E-PS, PU, CM, NB-S, BS, and GP: analyses and interpretation of data. E-PS and GP: drafting of the article. E-PS, PU, CM, NB-S, BS, BU, and GP: critical revision and editing and final approval of the article.

### Conflict of Interest

The authors declare that the research was conducted in the absence of any commercial or financial relationships that could be construed as a potential conflict of interest.

## Abbreviations

PSS:NICU: Parental Stressor Scale:Neonatal Intensive Care Unit
NICU: Neonatal intensive care unit.
